# Potential Negative Feedback between Age and Baseline Axial Length on Axial Elongation in High Myopia

**DOI:** 10.1016/j.xops.2025.100937

**Published:** 2025-09-04

**Authors:** Mariko Murata, Masahiro Miyake, Kenji Suda, Yuki Mori, Kazuya Morino, Wakako Okayama, Akitaka Tsujikawa

**Affiliations:** Department of Ophthalmology and Visual Sciences, Kyoto University Graduate School of Medicine, Kyoto, Japan

**Keywords:** Axial length elongation, GRS, High myopia

## Abstract

**Purpose:**

To evaluate the axial elongation in highly myopic eyes and assess the effects of age, sex, baseline axial length (AL), cataract surgery (CS), pathologic myopia (PM), baseline intraocular pressure (IOP), and genetic risk scores (GRSs).

**Design:**

Retrospective cohort study.

**Participants:**

This study included 614 eyes from 367 individuals with high myopia.

**Methods:**

The study assessed axial elongation rates and their associations with age, sex, baseline AL, CS, PM, and baseline IOP including potential interactions among these factors. Additionally, the study examined whether incorporating GRS improved the predictive model for axial elongation.

**Main Outcome Measures:**

Axial elongation rate in highly myopic eyes.

**Results:**

The study included 367 participants (217 [59.1%] females, 150 [40.9%] males) with a mean age of 58.9 ± 14.4 years and a mean AL of 28.6 ± 2.0 mm. The mean follow-up duration was 4.7 ± 2.7 years, and the average axial elongation rate was 0.031 ± 0.030 mm/year. Cataract surgery was associated with significantly slower axial elongation (*P* < 0.001). Multivariate analysis revealed that axial elongation increased with age and baseline AL but decreased with CS and an age–AL interaction. The best-fitting model excluded GRS, thus achieving a lower Akaike information criterion score (–573.4) than models including GRS.

**Conclusions:**

Axial elongation persists in highly myopic eyes (0.031 mm/year) but slows over time, owing to baseline AL–age interactions. Genetic risk scores have limited predictive utility in adulthood. This highlights the need for further research on genetic and environmental determinants of myopia progression.

**Financial Disclosure(s):**

Proprietary or commercial disclosure may be found in the Footnotes and Disclosures at the end of this article.

Axial length is a key parameter reflecting overall eye length, typically measuring approximately 24 mm in healthy eyes. A 1-mm increase in AL correlates with approximately 3 diopters of axial myopia and can exert mechanical stress on the choroid and optic nerve. Notably, a 1-mm increase in AL is associated with an odds ratio of 4.20 for developing myopic maculopathy, which is a leading cause of blindness in developed countries.^1^^,^^2^ Furthermore, the presence of myopia, including among children, is increasing globally.^3^, ^4^, ^5^ Therefore, understanding the causes of axial elongation is crucial.

In normal development, axial length (AL) increases during childhood, reaching an average of 22.4 mm by approximately 7 years of age and attaining adult levels by approximately 13 years.^6^^,^^7^ However, in high myopia, axial elongation may persist even in adulthood.^8^ Several studies have reported annual axial elongation rates in high myopia ranging from 0.01 to 0.06 mm/year.^9^, ^10^, ^11^, ^12^, ^13^, ^14^ Various factors have been extensively investigated, with baseline AL, female sex, age, posterior staphyloma, and other factors recognized as significant risk factors for axial elongation.^8^, ^9^, ^10^^,^^15^ However, previous reports have not examined the interactions between these risk factors, thus, highlighting the need for more sophisticated modeling to better understand axial elongation. Moreover, the role of genetic factors in longitudinal axial elongation is underexplored. Genome-wide association studies have identified multiple single nucleotide polymorphisms (SNPs) associated with AL, and the genetic risk scores (GRS) derived from these SNPs show strong correlations with AL.^16^, ^17^, ^18^ However, these associations have been established through cross-sectional studies, leaving the longitudinal relationship between GRS and axial elongation unexplored.

This study utilized data from 614 eyes of individuals with high myopia to evaluate the rate of axial elongation. We assessed the contributions of age, sex, baseline AL, cataract surgery (CS), pathologic myopia (PM), and baseline intraocular pressure (IOP) to axial elongation rate, including the potential interactions among these factors. Furthermore, we investigated whether incorporating GRS into the model improved predictive performance for axial elongation.

## Methods

This investigation is framed as a retrospective cohort study, approved by the Ethics Committee of Kyoto University Graduate School of Medicine, and conducted in accordance with the principles of the Helsinki Declaration. All the participants provided informed consent.

### Participants

We reviewed the medical records of 1262 patients who first presented to the Macular Clinic of Kyoto University Hospital between January 2011 and June 2018, including a total of 2524 eyes. The inclusion criteria comprised patients aged ≥20 years, with an initial AL ≥26 mm, who underwent at least two AL measurements, and the time between the first and last AL measurements was required to be >30 days. Eyes that underwent any intraocular surgical procedure—excluding CS—before or during the observation period were excluded from the analysis. Refraction measurements were performed using an autorefractor (ARK-530K, NIDEK), whereas the spherical equivalent power was calculated as the sum of the spherical power and half of the cylindrical power. Axial length and corneal curvature radius were measured using an IOLMaster 500 or 700 (Carl Zeiss Meditec) depending on the measurement period. We selected the measurement mode based on the lens status: phakic mode for phakia, pseudophakic mode for pseudophakic eyes, and aphakic mode for aphakia, ensuring that measurement errors were minimized. In this study, age was defined as the baseline age.

### Genetic Risk Score

To investigate whether axial elongation was associated with known genetic factors, we calculated the GRS for participants who were also included in the Kyoto High Myopia Cohort and had available genotype data. The details of the Kyoto High Myopia Cohort have been previously reported;^19^ therefore, only a brief overview is provided here.

Participants were genotyped using either Asian Screening Array-24 BeadChip or whole-genome sequencing. For the Asian Screening Array-24 BeadChip dataset, genotype quality control (QC) and imputation were performed, and only imputed SNPs with *R*^2^ ≥ 0.3 were included in the analysis. For the whole-genome sequencing dataset, SNPs that passed QC were included in the analysis. Further details on genotype imputation and QC for each dataset are provided in the Notes S1 (available at www.ophthalmologyscience.org).

Of the 31 susceptibility SNPs for AL previously reported in the Tohoku Medical Megabank Organization Eye Study of the Japanese population,^16^ those that passed the QC in all data sets were used to calculate the GRS. The GRS was computed using the following formula:

$$GRS=\sum_{SNPs}\left(\beta \times N_{EA}\right)\text{,}$$

where *β* represents the effect size, and *N*_*EA*_ denotes the number of effect alleles (0, 1, or 2) for each SNP included in the GRS calculation.

### Statistical Analysis

Estimates of the best linear unbiased predictors were calculated using a linear mixed-effects model based on longitudinal measurements of each eye and expressed in millimeters per year as the rate of axial elongation. In this model, we included random effects for eyes nested within participant identifier to account for participant-level differences and eye-level differences within participants. The analysis was conducted using SAS OnDemand for Academics (SAS Institute Inc).

Comparisons of axial elongation between sexes and between groups that underwent CS and those that did not were performed using the Mann–Whitney *U* test. Age and AL were also compared between sexes using the Mann–Whitney *U* test. AL, age, GRS, and baseline IOP were stratified into quartiles, and the Kruskal–Wallis test, followed by the Steel–Dunnett method, was employed to compare the rate of axial elongation. Additionally, myopic maculopathy, classified based on the Meta-Analysis for Pathologic Myopia (META-PM) classification, was analyzed using the Kruskal–Wallis test followed by the Steel–Dunnett method for each category. To identify the factors contributing to the prediction of axial elongation, multivariable generalized linear mixed models were used, with the axial elongation rate as the dependent variable and baseline AL, age, sex, CS during the observation period, baseline IOP, PM, and two-factor interactions as explanatory variables. A stepwise selection method was employed to identify the most relevant variables and construct the final model, with Akaike information criterion (AIC) used to assess model fit. Variance inflation factors, after mean centering, were assessed to evaluate multicollinearity among covariates. In addition, a Pearson correlation analysis was performed to evaluate potential multicollinearity between age and AL.

A generalized linear model analysis was utilized to evaluate whether incorporating GRS improved the predictive ability of the axial elongation model. In the analysis, the rate of axial elongation of the right eye was used as the dependent variable, with age, baseline AL, sex, CS, GRS, baseline IOP, PM, and two-factor interactions included as explanatory variables. For participants who did not have right eye axial elongation data but had left eye axial elongation data, the analysis was conducted using the rate of axial elongation of the left eye instead. A full model incorporating all variables and interaction terms was developed, and the optimal model was determined via stepwise selection based on the minimum AIC. Additionally, GRS was forced into the model, whereas a stepwise selection method was applied to derive the model with the lowest AIC.

To evaluate IOP before and after CS, we calculated the average IOP within 3 years before and after the surgery for 114 eyes with available IOP data that underwent CS during the observation period. Intraocular pressure measurements taken within 7 days after CS were excluded. The difference in mean IOP before and after CS was assessed using a Mann–Whitney *U* test. The association between the change in IOP and the axial elongation rate was examined using a linear mixed-effects model adjusting for age, baseline AL, and baseline IOP, while accounting for intereye correlation. Statistical analyses were conducted using RStudio (R version 4.3.2).

## Results

Table 1 presents the demographic and clinical characteristics of the patients. Axial elongation rates were calculated for 614 eyes from 367 participants, of whom 217 (59.1%) were females and 150 (40.9%) were males. The mean age was 58.9 ± 14.4 years, and the mean AL was 28.6 ± 2.0 mm. Females were significantly older than males (60.5 ± 14.1 vs 56.6 ± 14.6 years, *P*=0.001) and had a significantly longer baseline AL (28.7 ± 2.1 vs 28.3 ± 2.0 mm, *P* = 0.006). The average spherical equivalent was –10.0 ± 5.8 diopters and the mean corneal curvature was 7.7 ± 0.3 mm. The mean follow-up duration was 4.7 ± 2.7 years, and the average number of AL measurements was 3.01 ± 1.27 times. The mean IOP was 15.0 ± 3.7 mmHg. Out of the 614 eyes, 8 had unclear fundus photographs, resulting in classification for only 606 eyes. Among these 606 eyes classified by the category of myopic maculopathy, 317 eyes (52.3%) were categorized as category 0 or 1, 106 (17.5%) as category 2, 102 (16.8%) as category 3, and 81 (13.4%) as category 4.

**Table 1:** Patient Demographic Characteristics at Initial Examination

Baseline Demographics	Values	*P* Value
Patients (eyes), n	367 (614)	
Age, years total	58.9 (14.4)	
Male	56.6 (14.6)	**0.001**
Female	60.5 (14.1)	
Sex, female (%)	217 (59.1)	
Follow-up period, years	4.7 (2.7)	
AL, mm total	28.6 (2.0)	
Male	28.3 (2.0)	**0.006**
Female	28.7 (2.1)	
SE, D	–10.0 (5.8)	
Corneal curvature, mm	7.7 (0.3)	
IOP, mmHg	15.0 (3.7)	
Lens status, n (%)		
Phakia	506 (82.4)	
Pseudophakia	88 (14.3)	
Aphakia	20 (3.3)	
Category of myopic maculopathy, n (%)	606	
Category 0 or 1	317 (52.3)	
Category 2	106 (17.5)	
Category 3	102 (16.8)	
Category 4	81 (13.4)	

AL = axial length; D = diopter; SE = spherical equivalent; IOP = intraocular pressure.

Means (standard deviations) are presented.

*P* values <0.05 are indicated in bold.

Table 2 presents the rates of axial elongation stratified by various parameters. The mean axial elongation rate was 0.031 ± 0.030 mm/year. Males had a rate of 0.029 ± 0.022 mm/year, whereas females exhibited a rate of 0.032 ± 0.034 mm/year (*P* = 0.059). Comparisons across baseline AL quartiles revealed significantly higher rates in Q2 (26.90–28.05 mm), Q3 (28.05–29.79 mm), and Q4 (≥29.79 mm) than Q1 (26–26.90 mm). Further stratification by AL and age is detailed in the supplementary materials. Among the participants with ALs exceeding 27 mm, axial elongation was significantly faster than in those with lengths between 26 and 27 mm. However, no significant differences were observed when age was categorized into quartiles or 10-year increments as shown in Table S1 (available at www.ophthalmologyscience.org). Moreover, participants who underwent CS during the observation period, which included 119 eyes (5 of which remained aphakic without an intraocular lens after surgery), exhibited significantly slower axial elongation than those who did not (*P* < 0.001).

**Table 2:** Stratified Comparison of Axial Length Elongation Rates by Sex, Age, Baseline Axial Length, GRS, Cataract Surgery, Myopic Maculopathy, and Intraocular Pressure

	Group	Mean, mm/yr (SD)	*P* Value
Patients		0.031 (0.030)	
Sex			
Male		0.029 (0.022)	0.059
Female		0.032 (0.034)	
Age, yr			
<49.33	Q1	0.032 (0.020)	Reference
49.33–61.33	Q2	0.031 (0.019)	0.982
61.33–69.44	Q3	0.031 (0.045)	0.609
≥69.44	Q4	0.028 (0.027)	0.917
BAL, mm			
<26.90	Q1	0.024 (0.014)	Reference
26.90–28.05	Q2	0.028 (0.014)	**0.005**
28.05–29.79	Q3	0.036 (0.047)	**<0.001**
≥29.79	Q4	0.033 (0.030)	**<0.001**
GRS			
<–0.300	Q1	0.027 (0.014)	Reference
–0.300 to –0.164	Q2	0.037 (0.027)	0.483
–0.164 to –0.043	Q3	0.041 (0.08)	0.606
≥–0.043	Q4	0.030 (0.025)	0.989
Cataract surgery during the observation period			
Yes		0.025 (0.029)	**<0.001**
No		0.032 (0.030)	
Myopic maculopathy			
	0 or 1	0.0287 (0.018)	Reference
	2	0.0374 (0.023)	**<0.001**
	3	0.0339 (0.052)	0.215
	4	0.0248 (0.035)	0.990
Intraocular pressure			
<12.5	Q1	0.0287 (0.016)	Reference
12.5–15.0	Q2	0.0328 (0.046)	0.805
15.0–17.0	Q3	0.0297 (0.022)	0.952
≥17.0	Q4	0.0309 (0.027)	0.720

BAL = baseline axial length; GRS = genetic risk score; SD = standard deviation.

*P* values <0.05 are indicated in bold.

Table 3 presents the best multivariate analysis models with axial length growth rate as the dependent variable. The full model, which included all explanatory variables and two-factor interaction terms and is shown in Table S3 (available at www.ophthalmologyscience.org), had an AIC of –2295, whereas the best model achieved a lower AIC of –2525, thus indicating a better model fit. The AIC of the best model was more than 10 points lower, suggesting a significantly better performance than that of the full model.^20^ In the best model, age and AL were positively associated with axial elongation, while CS and the interaction between age and AL were negatively associated. Variance inflation factors after mean centering and correlation analyses indicated no concerning collinearity between age and AL. Detailed results are provided in the Table S4 (available at www.ophthalmologyscience.org). Notably, the effect of CS was statistically significant (*P* = 0.035), thereby suggesting its potential impact on axial elongation.

**Table 3:** Best Multivariate Analysis Models with Axial Length Growth Rate as the Dependent Variable

	Estimate	SE	*P* Value	AIC
Age	0.0040	0.0015	**0.010**	–2525
BAL	0.0098	0.0034	**0.004**	
CS	–0.0077	0.0037	**0.035**	
Age∗BAL	–0.0001	0.0001	**0.008**	

AIC = Akaike information criterion; BAL = baseline axial length; CS = cataract surgery; SE = standard error; ∗ = interaction term.

*P* values <0.05 are indicated in bold.

Among the 367 participants, genome-wide SNP data were available for 169 individuals, thus enabling the successful calculation of the GRS. Of the 31 susceptibility SNPs for AL, 28 were included in the GRS calculation. Details of these SNPs are provided in Table S2 (available at www.ophthalmologyscience.org). Table 4 compares the models. The full model, which included all explanatory variables and interaction terms, had an AIC of –548. When GRS was forcibly included in the model and stepwise selection was applied, the AIC improved to –572. However, the best model, derived through stepwise selection without forcing GRS, achieved the lowest AIC of –573. This indicates that incorporating the GRS did not enhance the predictive performance of the model for axial elongation for this adult cohort, as excluding GRS resulted in a better model fit.

**Table 4:** Comparison of Model Results: Best Model via Stepwise Method, Best Model with GRS Forced Inclusion via Stepwise Method, and Full Variable Model

	Model	AIC
Full model (including all variables and two-factor interaction terms)	(age + BAL + sex + CS + GRS + PM + IOP)^2^	–548.3
Best model with GRS forcibly incorporated into the model	age + BAL + CS + GRS + age∗CS + BAL∗CS	–571.6
Best model	age + BAL + CS + age∗CS + BAL∗CS	–573.4

AIC = Akaike information criterion; BAL = baseline axial length; CS = cataract surgery; GRS = genetic risk score; IOP = intraocular pressure; PM = pathologic myopia; ∗ = interaction term.

The preoperative mean IOP was 14.7 ± 2.9 mmHg, and the postoperative mean IOP was 14.3 ± 2.4 mmHg, with Mann–Whitney *U* test showing a significant difference (*P*=0.009). In multivariable linear mixed-effects analysis, adjusting for age, baseline AL, and baseline IOP, the change in IOP was not significantly associated with axial elongation rate (β = 0.00009, *P*=0.95).

## Discussion

In this study, we modeled the axial elongation rate in 614 eyes with high myopia using a linear mixed-effects model. In line with previous studies, we confirmed that longer ALs were associated with faster axial elongation. Notably, we identified an interaction between age and baseline AL. This indicated that while significant AL and older age are generally linked to increased axial elongation, the axial elongation rate slows as AL and age advance. Our findings suggest that incorporating the GRS did not enhance the predictive performance of the model for axial elongation in this adult longitudinal study.

Previous studies, primarily conducted in Asian populations, have reported that axial elongation in adults with high myopia ranges from approximately 0.01 to 0.06 mm/year.^9^, ^10^, ^11^, ^12^, ^13^, ^14^ The rate of AL elongation has been estimated using different methods. These include calculating the annualized AL change from 2 time points and applying linear mixed-effects models that incorporate data from ≥3 time points. The mixed-effects model is statistically robust because it accounts for both fixed and random effects while capturing individual variability, making it well-suited to our analysis. A recent large-scale study by Kong et al applied a mixed-effects model to analyze 1043 eyes and over 5359 data points. The study reported an axial elongation rate of 0.03 mm/year, which aligns with our findings and further supports the validity of our model.^21^ Therefore, our results suggest that an axial elongation rate of approximately 0.03 mm/year acts as a reasonable estimate.

Numerous studies have identified various factors influencing axial elongation in patients with high myopia. Baseline AL has been consistently recognized as a key determinant across studies, and our findings corroborate this trend.^9^, ^10^, ^11^, ^12^^,^^14^ Consequently, individuals with longer initial AL may be more susceptible to further elongation, thus warranting careful monitoring.

The role of sex in axial elongation remains controversial, with conflicting findings reported in the literature. Although several large-scale studies have identified female sex as a risk factor, our results did not support this association. In our study, although univariable analysis showed a trend toward a faster axial elongation rate in females compared with males (*P* = 0.059), sex was not retained as an independent factor in the multivariable model after stepwise selection. The observed effect of sex on axial elongation was likely confounded by baseline AL and age, as females had a significantly longer baseline AL and were significantly older than males. Previous reports indicated that 10% to 25% of participants exhibited axial elongation exceeding 0.1 mm/year, whereas only 1.1% of participants in our study showed this trend, thereby suggesting a potential impact on our findings.^10^^,^^21^ The variation in proportions may stem from differences in the study populations, including the younger age of participants in Kong et al's study and the more severe myopia in subjects analyzed by Ran et al. Both studies were based on hospital cohorts with myopia, which was also observed in our study. Future studies should include general population-based cohorts to better elucidate determinants of axial elongation.

The finding that CS is associated with a significantly slower axial elongation is novel and has not been previously reported. Previous studies have established that IOP decreases after CS in both normal and glaucomatous eyes.^22^, ^23^, ^24^, ^25^ Although the exact mechanism by which a reduced IOP influences axial elongation remains unclear, a leading hypothesis posits that a lower IOP may inhibit the activation of scleral fibroblasts, thereby suppressing scleral remodeling.^22^^,^^26^ Consequently, a reduction in IOP following CS may contribute to decreased axial elongation. In this study, we observed a reduction in mean IOP before and after CS. However, the association between the difference in preoperative and postoperative mean IOP and the axial elongation rate was not statistically significant, and it remains unclear whether the reduction in IOP truly suppresses axial elongation. Furthermore, it is important to consider that the intraocular lens implantation itself may also influence postoperative AL measurements, independent of the IOP reduction. This potential factor should be taken into account when interpreting the results. Therefore, the relationship between CS, IOP, and AL should be interpreted with caution, and further research is needed to clarify this association. Emerging evidence suggests that glaucoma eye drops may inhibit the progression of axial elongation, and randomized controlled trials are currently underway.^12^^,^^21^^,^^27^ Our findings may provide additional support for this hypothesis.

Modeling the interaction terms is crucial for understanding the axial elongation. Previous studies did not account for interaction terms in their models, implicitly suggesting a positive feedback mechanism in which an increased baseline AL corresponds to accelerated axial elongation. In contrast, our investigation of the interaction terms indicated a negative feedback mechanism, while the rate of axial elongation increased with a longer baseline AL and older age; their negative interaction gradually attenuated this acceleration. Based on this finding, AL does not continue to elongate indefinitely but is likely to plateau at a certain threshold. Since the orbit is a confined space, expansion of the eyeball may influence intraorbital pressure. The interaction between intraorbital pressure and IOP may play a role in restricting eyeball expansion.

The mechanisms underlying the axial elongation in adults with high myopia remain incompletely understood. Although previous studies have examined the risk factors for axial elongation, research on genetic determinants has been limited. Our finding that models excluding GRS exhibited a better fit than those incorporating it may initially appear to contradict existing literature demonstrating a strong association between GRS and AL.^16^ However, prior studies have primarily relied on cross-sectional analyses, whereas our study employs a longitudinal approach, making these findings not inherently contradictory. Specifically, while the GRS likely influences axial elongation during developmental stages and contributes to final AL, its impact on the elongation rate in adulthood appears minimal. Identifying genetic factors directly associated with axial elongation may enhance our understanding of myopia progression.

This study has some limitations. First, potential biases may arise from its hospital-based approach. While a general population cohort would be ideal, it may limit the sample size. A combined approach incorporating both hospital-based and general population cohorts could be more advantageous. Second, as most participants were Japanese, ethnic bias is a potential concern. Given that previous studies focused predominantly on Asian populations, future comparisons with White cohorts are essential. Third, SNPs not included in the GRS may also influence axial elongation in adulthood. As larger genome-wide association studies on AL become available, our findings may evolve.

Our findings indicate that while the axial elongation of highly myopic eyes persists at an average rate of 0.03 mm/year, this elongation is not indefinite. Instead, it gradually decelerates because of the interaction between baseline AL and age. Although genetic factors influence the final AL, our results reveal that the GRS has limited predictive value for axial elongation in adulthood. Identifying additional genetic and environmental determinants may enhance our understanding of myopia progression.

## Supplementary Data

Table S4

Note S1
