# Supplementary material for: Potential Negative Feedback between Age and Baseline Axial Length on Axial Elongation in High Myopia
Source: Ophthalmol Sci. 2025 Sep 4;6(1):100937. doi: 10.1016/j.xops.2025.100937 (PMC12547896; doi:10.1016/j.xops.2025.100937)
Supplement: Table S1 [file mmc1.pdf]

Supplementary Table 1. Comparison of axial elongation rates by detailed stratification of axial length and age

| | | **Eyes (n)** | **Mean, mm/year (SD)** | ***p*-value** |
|---|---|---|---|---|
| Axial length, mm | 26–27 | 170 | 0.024 (0.013) | reference |
| | 27–28 | 134 | 0.028 (0.014) | **0.04** |
| | 28–29 | 98 | 0.032 (0.019) | **<0.001** |
| | 29–30 | 68 | 0.044 (0.067) | **<0.001** |
| | >30 | 144 | 0.032 (0.030) | **<0.001** |
| | | | | |
| Age, years | 20–30 | 22 | 0.035 (0.023) | reference |
| | 30–40 | 58 | 0.039 (0.024) | 0.82 |
| | 40–50 | 86 | 0.026 (0.013) | 0.16 |
| | 50–60 | 118 | 0.031 (0.020) | 0.91 |
| | 60–70 | 181 | 0.030 (0.043) | 0.60 |
| | 70–80 | 118 | 0.030 (0.026) | 0.90 |
| | >80 | 31 | 0.027 (0.021) | 0.38 |

P values < 0.05 are indicated in bold.

SD, standard deviation
