# Supplementary material for: Potential Negative Feedback between Age and Baseline Axial Length on Axial Elongation in High Myopia
Source: Ophthalmol Sci. 2025 Sep 4;6(1):100937. doi: 10.1016/j.xops.2025.100937 (PMC12547896; doi:10.1016/j.xops.2025.100937)
Supplement: Table S2 [file mmc2.pdf]

Supplementary Table 2. SNPs used for the calculation of genetic risk scores

Chr	Lead SNP	Position	Nearest Gene	RA	EA	EA Freq	*B*	*SE*
15	rs16959560	35006600	*LINC02252–GJD2*	A	G	0.535	0.084	0.009
17	rs151278468	54634412	*ANKFN1–NOG*	G	A	0.012	-0.397	0.044
7	rs141313179	158906675	*VIPR2*	A	G	0.010	0.364	0.040
22	rs10453459	46366127	*WNT7B*	G	C	0.668	-0.085	0.009
11	rs7936359	40148976	*LRRC4C*	T	A	0.204	0.078	0.011
15	rs13380109	79378775	*RASGRF1*	G	A	0.513	0.06	0.009
2	rs77311538	233386148	*PRSS56*	C	G	0.093	-0.103	0.015
4	NA	17883603	*FAM184B–DCAF16 –LCORL*	GA	GAA	0.231	-0.072	0.011
22	rs4823003	29410232	*ZNRF3*	A	C	0.595	-0.058	0.009
14	rs10459508	54485490	*BMP4–CDKN3*	A	G	0.521	-0.056	0.009
16	rs4889024	79796196	*MAFTRR*	C	A	0.716	-0.062	0.010
6	rs7744813	73643289	*KCNQ5*	C	A	0.763	0.061	0.010
12	rs3138142	56115585	*RDH5*	C	T	0.049	-0.117	0.021
10	rs11204213	48388228	*RBP3*	C	T	0.018	0.188	0.033
8	rs16890057	40726582	*ZMAT4*	G	A	0.068	-0.1	0.017
16	rs3848363	370484	*AXIN1*	T	C	0.405	-0.05	0.009
8	rs36005291	60179048	*LOC100505501–CA8*	CA	C	0.442	-0.048	0.009
3	rs7651194	47540917	*ELP6*	G	A	0.551	-0.047	0.009
20	rs146314970	6757519	*BMP2*	T	TA	0.185	0.059	0.011
6	rs58353542	170491975	*LOC102724511– LOC154449*	T	G	0.128	-0.062	0.013
13	NA	50134748	*RCBTB1*	AT	ATT	0.124	-0.063	0.013
11	rs3741210	2169540	*IGF2-AS*	A	G	0.454	-0.042	0.009
4	rs147732642	55089323	*GSX2–PDGFRA*	C	CT	0.152	-0.055	0.012
15	rs965480	77781926	*HMG20A– LOC101929457*	A	G	0.407	0.04	0.009
8	rs72609833	121669010	*SNTB1*	C	A	0.375	0.039	0.009
10	rs7902218	104740210	*CNNM2*	A	G	0.285	-0.043	0.010
5	rs35305813	151225351	*GLRA1*	T	TA	0.104	0.061	0.014
2	rs60806750	36577213	*MIR548AD–CRIM1-DT*	T	C	0.330	0.036	0.009

The positions were based on the National Center for Biotechnology Information Build 37.
SNP, single-nucleotide polymorphism; CHR, chromosome; BP, base pair; RA, reference allele; EA, effect allele; EAF, effect allele frequency; SE, standard error.
