# Supplementary material for: Potential Negative Feedback between Age and Baseline Axial Length on Axial Elongation in High Myopia
Source: Ophthalmol Sci. 2025 Sep 4;6(1):100937. doi: 10.1016/j.xops.2025.100937 (PMC12547896; doi:10.1016/j.xops.2025.100937)
Supplement: Table S3 [file mmc3.pdf]

Supplementary Table 3. Full multivariable analysis model with axial length growth rate as the dependent variable

	Full model		
	**Estimate**	**SE**	***p*-value**
Age	0.0034	0.0017	0.053
Sex	0.0211	0.0496	0.671
BAL	0.0156	0.0053	**0.003**
CS	0.0748	0.0611	0.222
PM	0.0520	0.0558	0.352
IOP	0.0060	0.0046	0.198
Age*Sex	-0.0001	0.0002	0.654
Age*BAL	-0.0001	0.0001	0.060
Age*CS	-0.0004	0.0004	0.300
Age*PM	0.0000	0.0003	0.876
Age*IOP	0.0000	0.0000	0.626
Sex*BAL	-0.0009	0.0018	0.599
Sex*CS	0.0026	0.0078	0.740
Sex*PM	0.0030	0.0070	0.666
Sex*IOP	0.0002	0.0008	0.817
BAL*CS	-0.0017	0.0019	0.357
BAL*PM	-0.0017	0.0018	0.351
BAL*IOP	-0.0002	0.0002	0.168
CS*PM	-0.0051	0.0082	0.530
CS*IOP	0.0000	0.0010	0.975
PM*IOP	-0.0001	0.0009	0.934
AIC	-2295		

BAL, baseline axial length; CS, cataract surgery; PM, pathologic myopia; IOP, intraocular pressure; SE, standard error; AIC, Akaike information criterion.
