# Supplementary material for: Potential Negative Feedback between Age and Baseline Axial Length on Axial Elongation in High Myopia
Source: Ophthalmol Sci. 2025 Sep 4;6(1):100937. doi: 10.1016/j.xops.2025.100937 (PMC12547896; doi:10.1016/j.xops.2025.100937)
Supplement: Table S4 [file mmc4.pdf]

Supplementary Table 4. Multicollinearity and correlation in the best model

Metric	Value
Age (centered) VIF	1.061
Axial length (centered) VIF	1.048
Age (centered) ＊ axial length (centered) interaction VIF	1.023
Cataract surgery VIF	1.072
Pearson correlation (age, AL)	0.108

VIF, variance inflation factor; AL, axial length.

Interpretation:

All VIF values after mean centering were approximately 1, indicating no multicollinearity among the predictors. The correlation between age and AL was statistically significant but weak.
