# Supplementary material for: Potential Negative Feedback between Age and Baseline Axial Length on Axial Elongation in High Myopia
Source: Ophthalmol Sci. 2025 Sep 4;6(1):100937. doi: 10.1016/j.xops.2025.100937 (PMC12547896; doi:10.1016/j.xops.2025.100937)
Supplement: Note S1 [file mmc5.pdf]

Supplementary Methods

Methodological details

Details of the Kyoto High Myopia Cohort

The Kyoto High Myopia cohort comprised patients with high myopia recruited from the Kyoto University Hospital, Tokyo Medical and Dental University, Fukushima Medical University Hospital, Ozaki Eye Hospital, and Kobe City Medical Center General Hospital as previously described.[1–6] All participants in the Kyoto High Myopia Cohort underwent dilated indirect and contact lens slit-lamp biomicroscopy, automatic objective refraction evaluation, measurement of the axial length by applanation A-scan ultrasonography (UD-6000; Tomey, Nagoya, Japan) or partial coherence interferometry (IOLMaster; Carl Zeiss Meditec, Dublin, CA), color fundus imaging and spectral-domain OCT with or without OCT angiography fundus autofluorescence, fluorescein angiography, and/or indocyanine green angiography. Participants were genotyped using the Illumina HumanHap550 BeadChip, Illumina Human660W-Quad BeadChip, Illumina Infinium Asian Screening Array-24 BeadChip, or whole genome sequencing.

All procedures were performed in accordance with the Declaration of Helsinki after approval of the protocols by the Institutional Review Board and the Ethics Committee of each participating institute. The purpose and procedures of the study were fully disclosed to all participants, and written informed consents were obtained from them.

Genotyping and Imputations

The ASA dataset

Genomic DNA was prepared from peripheral blood samples for SNP genotyping, according to standard laboratory procedures. After genome-wide SNP genotyping, stringent initial quality control (QC) was performed on the data of participants and SNPs. Through the QC process, (1) participants with a call rate < 0.9, (2) SNPs with a call rate < 0.9, (3) SNPs with a minor allele frequency (MAF) < 0.01, and (4) SNPs showing deviation from the Hardy-Weinberg equilibrium (HWE; $P < 1.0 \times 10^{-6}$ in control subjects) were excluded.

After the initial QC, genomic imputation was performed for each dataset using the Michigan imputation server (https://imputationserver.sph.umich.edu/index.html#!pages/home), with the 1000 Genomes dataset (phase3 v5 release) of East Asians as a reference panel. Subsequently, we conducted secondary QC. We excluded (1) imputed SNPs for which R^2 was <0.9, (2) SNPs with MAF < 0.01, and (3) SNPs showing deviation from the HWE ($P < 1.0 \times 10^{-6}$ in control subjects).

The WGS dataset

For 274 samples from the Kyoto High Myopia Cohort, DNA from whole-blood samples was sequenced on the Illumina HiSeq X platform using the PCR-free 150 paired-end of 450 base pair fragments protocol. The sequenced raw data were processed, and a FASTQ file was obtained. For

these samples, FASTQ data were aligned with bwa ver. 0.7.17 and the "mem" option to GRCh38DH.fa, and a joint-call operation was conducted with the GATK best practices workflow (GATK ver. 4.1.4). To stabilize the joint calling accuracy and consistent variant calls to the control dataset, we used 11,238 WGS datasets,[7] and extracted the variant call format file of eligible samples. From this dataset, we selected participants who met the inclusion criteria.

Finally, we applied the following QC; we excluded (1) SNPs with a call rate < 0.9, (2) SNPs with MAF < 0.01, (3) SNPs showing deviation from the HWE ($P < 1.0 \times 10^{-6}$ in control subjects), and (4) participants with a call rate < 0.9.

References

1. Hosoda Y, Yoshikawa M, Miyake M, Tabara Y, Shimada N, Zhao W, Oishi A, Nakanishi H, Hata M, Akagi T, Ooto S. CCDC102B confers risk of low vision and blindness in high myopia. *Nat Commun.* 2018;9(1):1782.

2. Miyake M, Yamashiro K, Nakanishi H, Nakata I, Akagi-Kurashige Y, Kumagai K, Oishi M, Tsujikawa A, Moriyama M, Ohno-Matsui K, Mochizuki M. Evaluation of pigment epithelium-derived factor and complement factor I polymorphisms as a cause of choroidal neovascularization in highly myopic eyes. *Invest Ophthalmol Vis Sci.* 2013;54(6):4208-4212.

3. Nakanishi H, Gotoh N, Yamada R, Yamashiro K, Otani A, Hayashi H, Tsujikawa A, Shimada N, Ohno-Matsui K, Mochizuki M, Saito M. ARMS2/HTRA1 and CFH polymorphisms are not associated with choroidal neovascularization in highly myopic eyes of the elderly Japanese population. *Eye.* 2010;24(6):1078-1084.

4. Nakanishi H, Yamada R, Gotoh N, Hayashi H, Yamashiro K, Shimada N, Ohno-Matsui K, Mochizuki M, Saito M, Iida T, Matsuo K. A genome-wide association analysis identified a novel susceptible locus for pathological myopia at 11q24.1. *PLoS Genet.* 2009;5(9):e1000660.

5. Akagi-Kurashige Y, Kumagai K, Yamashiro K, Nakanishi H, Nakata I, Miyake M, Tsujikawa A, Moriyama M, Ohno-Matsui K, Mochizuki M, Yamada R. Vascular endothelial growth factor gene polymorphisms and choroidal neovascularization in highly myopic eyes. *Invest Ophthalmol Vis Sci.*

2012;53(4):2349-2353.

6. Morino K, Miyake M, Nagasaki M, et al. Genome-wide Meta-analysis for Myopic Macular Neovascularization Identified a Novel Susceptibility Locus and Revealed a Shared Genetic Susceptibility with Age-Related Macular Degeneration. Ophthalmol Retina. Published online November 1, 2024.

7. Nagasaki M, Sekiya Y, Asakura A, Teraoka R, Otokozawa R, Hashimoto H, Kawaguchi T, Fukazawa K, Inadomi Y, Murata KT, Ohkawa Y. Design and implementation of a hybrid cloud system for large-scale human genomic research. *Hum Genome Var.* 2023;10(1):6.
